# Consensus for the management of analgesia, sedation and *delirium* in adults with COVID-19-associated acute respiratory distress syndrome

**DOI:** 10.5935/0103-507X.20210005

**Published:** 2021

**Authors:** Manuel Donato, Federico Carlos Carini, María Julia Meschini, Ignacio López Saubidet, Adela Goldberg, Marisol García Sarubio, Daniela Olmos, Rosa Reina

**Affiliations:** 1 Hospital General de Agudos José María Penna - Buenos Aires, Argentina.; 2 Ministerio de Salud de la Nación Argentina - Buenos Aires, Argentina.; 3 Instituto de Efectividad Clínica y Sanitaria - Buenos Aires, Argentina.; 4 Hospital Italiano de Buenos Aires - Buenos Aires, Argentina.; 5 Hospital Interzonal General de Agudos General San Martín - La Plata, Argentina.; 6 Centro de Educación Médica e Investigaciones Clínicas “Norberto Quirno” - Buenos Aires, Argentina.; 7 Sanatorio de La Trinidad Mitre - Buenos Aires, Argentina.; 8 Hospital Municipal Príncipe de Asturias - Córdoba, Argentina.

**Keywords:** COVID-19, SARS-CoV-2, Pain, Analgesia, Deep sedation: Delirium, Respiration, artificial, COVID-19, SARS-CoV-2, Dolor, Analgesia, Sedación profunda: *Delirium*, Respiración artificial

## Abstract

**Objective:**

To propose agile strategies for a comprehensive approach to analgesia, sedation, *delirium,* early mobility and family engagement for patients with COVID-19-associated acute respiratory distress syndrome, considering the high risk of infection among health workers, the humanitarian treatment that we must provide to patients and the inclusion of patients’ families, in a context lacking specific therapeutic strategies against the virus globally available to date and a potential lack of health resources.

**Methods:**

A nonsystematic review of the scientific evidence in the main bibliographic databases was carried out, together with national and international clinical experience and judgment. Finally, a consensus of recommendations was made among the members of the Committee for Analgesia, Sedation and *Delirium of the Sociedad Argentina de Terapia Intensiva*.

**Results:**

Recommendations were agreed upon, and tools were developed to ensure a comprehensive approach to analgesia, sedation, *delirium,* early mobility and family engagement for adult patients with acute respiratory distress syndrome due to COVID-19.

**Discussion:**

Given the new order generated in intensive therapies due to the advancing COVID-19 pandemic, we propose to not leave aside the usual good practices but to adapt them to the particular context generated. Our consensus is supported by scientific evidence and national and international experience and will be an attractive consultation tool in intensive therapies.

## Abstract (es)

**Objetivo:**

Proponer estrategias agile para este abordaje integral de la analgesia, sedación, *delirium,* implementación de movilidad temprana e inclusión familiar del paciente con síndrome de dificultad respiratoria aguda por COVID-19, considerando el alto riesgo de infección que existe entre los trabajadores de salud, el tratamiento humanitario que debemos brindar al paciente y su familia, en un contexto de falta estrategias terapéuticas específicas contra el virus globalmente disponibles a la fecha y una potencial falta de recursos sanitarios.

**Metodos:**

Se llevó a cabo una revision no sistemática de la evidencia científica en las principales bases de datos bibliográficos, sumada a la experiencia y juicio clínico nacional e internacional. Finalmente, se realizó un consenso de recomendaciones entre los integrantes del Comité de Analgesia, Sedación y *Delirium* de la Sociedad Argentina de Terapia Intensiva.

**Resultados:**

Se acordaron recomendaciones y se desarrollaron herramientas para asegurar un abordaje integral de analgesia, sedación, *delirium,* implementación de movilidad temprana e inclusión familiar del paciente adulto con síndrome de dificultad respiratoria aguda por COVID-19.

**Discusión:**

Ante el nuevo orden generado en las terapias intensivas por la progresión de la pandemia de COVID-19, proponemos no dejar atrás las buenas prácticas habituales, sino adaptarlas al contexto particular generado. Nuestro consenso está respaldado en la evidencia científica, la experiencia nacional e internacional, y será una herramienta de consulta atractiva en las terapias intensivas.

## INTRODUCTION

Coronavirus disease 2019 (COVID-19) is a human respiratory pathology caused by infection with the novel coronavirus identified by the acronym SARS-CoV-2.^(1)^ On March 11, 2020, the World Health Organization (WHO) declared COVID-19 a pandemic, and since that time until October 10, approximately 36,754,395 confirmed cases and 1,064,838 deaths have been reported worldwide.^(2)^ As of that date, 1,262,476 confirmed cases and 34,183 deaths have been reported in Argentina, which is higher than in most Latin American countries.^(2,3)^

There is no specific drug against this virus or a globally available vaccine. Although dexamethasone and hydrocortisone have been shown to improve survival in severe cases of COVID-19 and heparins play an important role in preventing deep vein thrombosis (also in severe cases), currently the best strategy to deal with the pandemic is prevention of infection through public policy measures.^(4)^ The incubation period of SARS-CoV-2 infection is 2 to 14 days, and most infections are spread person to person, being highly transmissible.^(5)^ The Brigham and Women’s Hospital, Division of General Internal Medicine, of Harvard Medical School has proposed a clinical-therapeutic classification of the disease that divides the course of the disease into different stages and in turn identifies 2 overlapping but different pathological subsets: the first triggered by the virus and the second by the host’s response to the virus.^(6)^ Stage I or mild disease occurs at the time of inoculation and early establishment of the disease; Stage II occurs when lung compromise is established in the person; and Stage III or severe disease manifests as extrapulmonary systemic hyperinflammation.

The increased morbidity and mortality due to COVID-19 is largely due to acute viral pneumonitis that progresses to acute respiratory distress syndrome (ARDS).^(7)^ Some reports suggest that up to 20% of infected people develop serious disease that requires hospitalization, with most cases in elderly patients with comorbidities (obesity, diabetes, chronic kidney disease, hypertension, heart disease and chronic lung disease).^(5,6)^ It is estimated that between 5 and 8% of those infected require admission to an intensive care unit (ICU).^(7-11)^ In our country, an early public policy gained time for the health system to prepare in terms of its response capacity for the pandemic, managing to increase in particular the supply of necessary hospital supplies and 12,450 beds, almost 50% more than in pre-pandemic ICU conditions.^(12,13)^ However, recently, the Argentine health system has become progressively and dangerously more saturated, explained in part by a significant increase in ICU admissions in all provinces where there was practically no community circulation of the virus. This fact has made necessary the application of various protocols in the ICU, mainly due to the exponential increase in the use of health resources and care to reduce the risk of contagion in health personnel.

**Figure 1:**
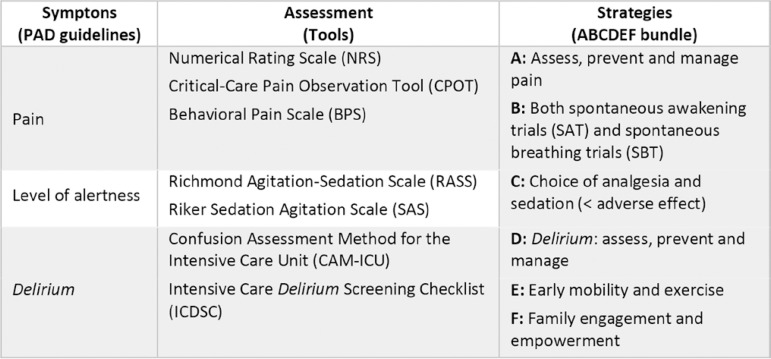
Components of the ABDCEF bundle.^(14)^

In the ICU, the comprehensive approach to achieve comfort, safety and facilitate interventions for life support for critical patients with COVID-19 ARDS mainly includes the systematic assessment of analgesia, sedation and *delirium* (ASD) in critical care, in addition to early mobility and family engagement. All this is reflected in the 2018 clinical practice guidelines for the prevention and management of pain, agitation/sedation, *delirium*, immobility and sleep disruption (PADIS) in adult patients in the ICU and the ABCDEF bundle of measures (Figure 1), published by the Society of Critical Care Medicine.^(14,15)^ Severe ARDS produced by SARS-CoV-2 during the COVID-19 pandemic challenged our ability to create, adapt and maintain work protocols, such as those proposed in the ABCDEF bundle. Additionally, the best available evidence for the management of these patients came from high-income countries with better-prepared health systems, where protocols usually have a high adoption rate and there is usually no lack of health resources.

The objective of this consensus is to propose targeted strategies and tools for the optimal management of ASD in this population, taking into account the high risk of contagion that exists among health workers, the humanitarian treatment we must provide to patients and the inclusion of patients’ families but in a context of a lack of specific globally available therapeutic strategies against the virus and a potential lack of health resources that could occur when the health system is saturated.^(16)^

## METHODS

This document was produced by consensus of nominal groups. The document obtained was based on a nonsystematic review of the scientific evidence, added to the judgment and clinical experience of the group of participating experts and other groups throughout the world.

The literature searches designed by the authors were performed in the Cochrane Database of Systematic Reviews (CDSR), the Cochrane Central Register of Controlled Trials (CENTRAL), the Database of Abstracts of Reviews of Effects (DARE), MEDLINE, and EMBASE. The basic search strategy designed for Medline (PubMed) included the following terms. Analgesia: (Coronavirus[Mesh] OR Spike glycoprotein, COVID-19 virus[Supplementary Concept] OR Severe Acute Respiratory Syndrome Coronavirus 2[Supplementary Concept] OR COVID-19[Supplementary Concept] OR Corona Virus[tiab] OR COVID-19[tiab] OR COVID19[tiab] OR 2019-nCoV[tiab] OR SARS-CoV-2[tiab] OR SARS-CoV2[tiab] OR (Pneumonia[tiab] AND Wuhan[tiab] AND 2019[tiab]) OR (Coronavir*[tiab] AND 2019[tiab])) AND (Critical Care[Mesh] OR Care, Critical OR Intensive Care[tiab] OR Care, Intensive[tiab]) AND (Analgesic[tiab] OR Analgesic*[tiab] OR Anodynes[tiab] OR Antinociceptive Agents[tiab]). Sedación: (Coronavirus[Mesh] OR Spike glycoprotein, COVID-19 virus[Supplementary Concept] OR Severe Acute Respiratory Syndrome Coronavirus 2[Supplementary Concept] OR COVID-19[Supplementary Concept] OR Corona Virus[tiab] OR COVID-19[tiab] OR COVID19[tiab] OR 2019-nCoV[tiab] OR SARS-CoV-2[tiab] OR SARS-CoV2[tiab] OR (Pneumonia[tiab] AND Wuhan[tiab] AND 2019[tiab]) OR (Coronavir*[tiab] AND 2019[tiab])) AND (Critical Care[Mesh] OR Care, Critical OR Intensive Care[tiab] OR Care, Intensive[tiab]) AND (Hypnotics and Sedatives[MeSH] OR Sedatives and Hypnotic*[tiab] OR Hypnotic*[tiab] OR Sedative*[tiab]). *Delirium*: (Coronavirus[Mesh] OR Spike glycoprotein, COVID-19 virus[Supplementary Concept] OR Severe Acute Respiratory Syndrome Coronavirus 2[Supplementary Concept] OR COVID-19[Supplementary Concept] OR Corona Virus[tiab] OR COVID-19[tiab] OR COVID19[tiab] OR 2019-nCoV[tiab] OR SARS-CoV-2[tiab] OR SARS-CoV2[tiab] OR (Pneumonia[tiab] AND Wuhan[tiab] AND 2019[tiab]) OR (Coronavir*[tiab] AND 2019[tiab])) AND (Critical Care[Mesh] OR Care, Critical OR Intensive Care[tiab] OR Care, Intensive[tiab]) AND (*Delirium* [MeSH] OR Delirium*[tiab]).

The inclusion of systematic reviews randomized controlled clinical trials and clinical practice guidelines was prioritized. The Editorial Board included intensivist physicians, pharmacists and kinesiologists who addressed a protocol for managing ASD in adults with ARDS caused by COVID-19. We will consider the different stages that critical patients go through, from the initial approach to the airway, to mechanical ventilation approaches in the different phases and to the withdrawal process. For each stage, the mentioned sources of bibliographic information were analyzed, and recommendations were established.

A group of independent experts formed the Review Committee. This group analyzed the document and suggested revisions, which were discussed until reaching a final consensus.

## RESULTS

### Rapid sequence of orotracheal intubation in adults with ARDS caused by COVID-19

**We recommend** orotracheal intubation (OTI) only in adults with COVID-19 and moderate to severe respiratory impairment who present increased respiratory effort with a respiratory rate greater than 30rpm and arterial oxygen partial pressure/fraction of inspired oxygen (PaO_2_/FiO_2_) less than 200 with an FiO_2_ greater than 50%. An algorithm adapted by members of the *Sociedad Argentina de Terapia Intensiva* for orotracheal intubation is proposed.

**Figure 2:**
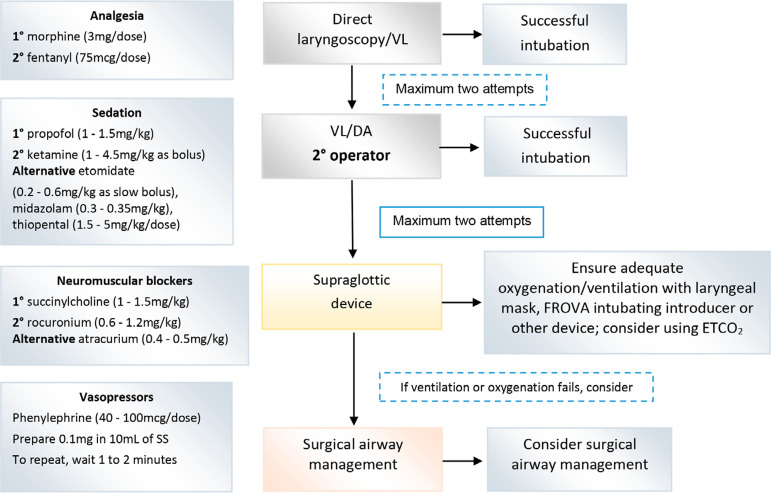
Sequence for the adapted orotracheal intubation sequence.^(22)^

Several health centers worldwide have reported that the majority of patients with COVID-19 ARDS have required intubation within the first 24 hours of being admitted to the ICU and within less than 8 hours for patients with associated risk factors, who require prolonged periods of mechanical ventilation, i.e., 3 to 4 weeks, with very high mortality.^(17-19)^ Therefore, the decision to intubate should always be made taking into account these outcomes and having assessed the potential recoverability of the patient. The risk of aerosolization during any maneuver in the airway is high to very high and requires the use of maximum protection personal protective equipment (N95 - type mask, isolation gown, gloves, eye protection and face mask) always accompanied by adequate hand hygiene.^(20,21)^ It is recommended to use fast-acting drugs to reduce the need for bag-valve-mask ventilation and the consequent risk of generating aerosolization of the patient’s secretions. The plan should always include, and in the next order, analgesia, sedation and neuromuscular blockers (NMBs). An alternative is the use of ketamine, which has analgesic and sedative effects; where the use of opioids could be avoided. Figure 2 summarizes the protocol we propose for this maneuver, with a useful format to use as a checklist at bedside.^(22)^

SS - 0.9% saline solution; VL - videolaryngoscopy; DA - difficult airway; ETCO2 – end-tidal carbon dioxide.

### Assessment and monitoring tools

#### Pain

**We recommend** systematically using the Behavioral Pain Scale (BPS) or the Critical-Care Pain Observation Tool (CPOT) in noncommunicative adults based on the developmental phase of COVID-19 ARDS.

**We recommend**, regardless of the developmental phase or depth of sedation, in these patients achieving and maintaining an analgesia target < 5 on the BPS and < 3 on the CPOT.

The best strategy to achieve relevant clinical outcomes in patients while preserving first-line drugs is to comply with the ABCDEF bundle.^(14)^ The systematic and ongoing assessment of pain, agitation and *delirium* in adults with COVID-19 ARDS is the most effective, safe, fast and inexpensive measure to preserve drugs. That is why we highlight its importance effectively in this document in our language to have at the patient’s bedside.

Based on the patient’s ability to communicate, pain reporting scales, such as the numerical rating scale (NRS), can be used in communicative patients, or behavior-based observation scales, such as the BPS and the CPOT, can be used when a patient cannot communicate.^(14,23)^ However, none can be applied to deeply sedated patients, defined according to the Richmond agitation-sedation scale (RASS) as -3 to -5, or with NMBs; limiting their use in adults with moderate/severe COVID-19 ARDS.^(15)^ Additionally, in these cases, we recommend starting and always maintaining preventive analgesia.

**Figura 3:**
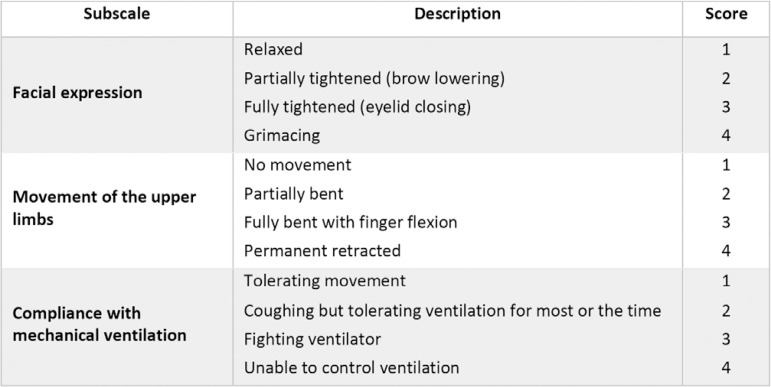
Behavioral pain scale.^(24)^

The BPS tool assesses 3 subscales, i.e., facial expression, upper limb movement and compliance with mechanical ventilation, and can be used in patients in deep sedation who are unable to express themselves (Figure 3).^(24)^ Each subscale is scored from 1 to 4, for a possible total score ranging from 3 to 12.

**Figure 4:**
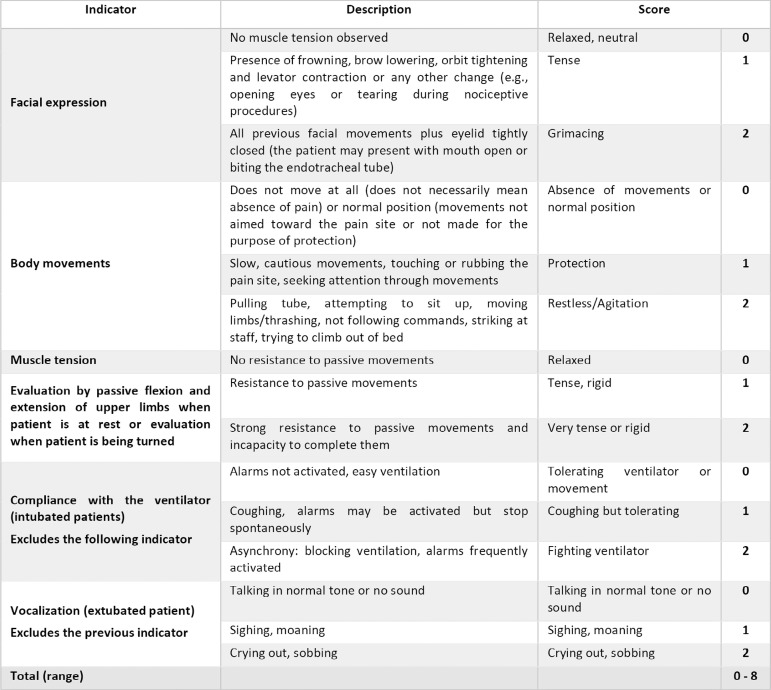
Critical-care pain observation tool.^(25)^

The CPOT, on the other hand, is based on scores of 4 components: facial expression; body movements; compliance with the ventilator (or vocalization for extubated patients); and muscle tension.^(25)^ Each component is scored from 0 to 2, with a possible total score ranging from 0 and 8 (Figure 4). Its advantage is that it scores the intensity of behavioral reactions of the patient and not the intensity of pain itself; it also allows assessments of patients who cannot self-report.

In the case of communicative patients, pain can be assessed with a response to a simple question (Are you in pain? Yes/No) or by rating pain intensity using a scale from 0 (absence of pain) to 10 (maximum pain imaginable). Both the NRS and subjective perception can be used equally.^(26)^

#### Agitation or level of alertness

**We recommend** the systematic use of the RASS in adults by goals according to the developmental phase of COVID-19 ARDS:

- Early phase or moderate/severe ARDS (deep sedation): Target RASS sedation level -4/-5. Given the availability of the processed electroencephalogram (pEEG), we always recommend their use during this phase with a bispectral index scale (BIS^©^) target between 40 and 60;
- Intermediate phase or mild ARDS (light sedation): Target RASS sedation level 0 to -3. At these levels of sedation, the use of pEEG may not be necessary; if it is used, we recommend a BIS^©^ target between 60 and 80; and
- Advanced phase or weaning (light sedation or absence of sedation): Target RASS sedation level 1 to -1.

**Figure 5:**
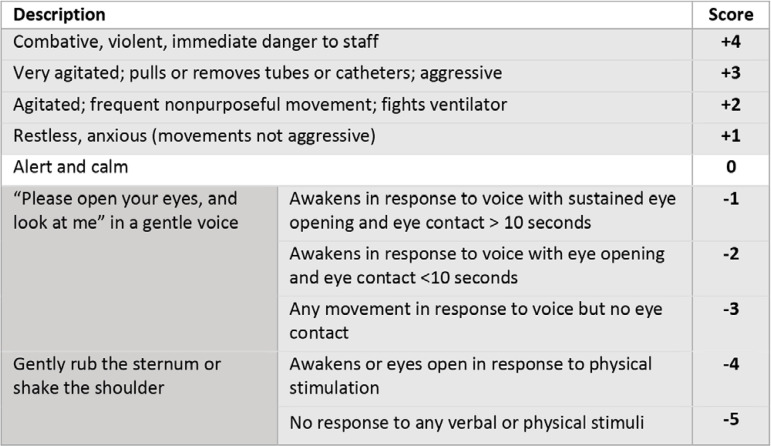
Richmond agitation-sedation scale.^(29)^

A Cochrane systematic review with meta-analysis published in 2018 showed that the implementation of sedation protocols in adults and infants in the ICU was not superior to the usual care practices for mortality, length of mechanical ventilation and length of hospital stay.^(27)^ The absence of high-quality evidence in support of a specific protocol led to opinion-based approaches.^(28)^ Despite this, frequent and constant monitoring of the level of alertness with validated tools is ideal in the ICU. However, due to the high risk of infection of health workers and the usual need for deep sedation of patients with COVID-19 infection, this task is difficult. We propose using validated tools depending on the clinical phase of the patient and taking into account the sedation levels and the requirement of NMBs. Through RASS (Figure 5), a subjective assessment can be made with 10 possible values, with positive values corresponding to different levels of agitation, and negative values corresponding to sedation.^(29,30)^

The objective assessment through EEG-based anesthetic depth monitors is based on measuring brain electrical activity in 2 or 4 derivatives.^(31)^ According to its limited availability, its use is only proposed for patients under deep sedation (RASS -4/-5) where clinical assessment is not possible. For its use, the following recommendations are provided: ensure that the electrodes are properly placed, allow at least 3-5 minutes to achieve a stable signal, look for the presence of artifacts, and assess the signal quality, the activity of the electromyogram, the level of sedation (the target should be between 40 and 60 in the BIS^©^ monitor and CoNox^TM^, at a lower value the sedation will be deeper), the suppression rate (as close to 0 as possible) and the EEG wave to confirm that the readings are appropriate for each isolated value. We recommend reviewing the recommendations of the *International Consortium for EEG Training of Anesthesia Practitioners*. Additionally, it also stands out the Riker Sedation-Agitation Scale (SAS) that consists of a subjective assessment with 7 individual tiers, with a score of 5 - 7 corresponding to agitation, 1-3 corresponding to sedation, and 4 corresponding to a calm and cooperative patient. Unlike the RASS, the Riker SAS assesses the response to painful stimuli.^(32)^ Finally, subjective assessment allows assessing the patient’s response to various stimuli: auditory, tactile or painful; however, such assessments cannot be used when the patient is under the effects of NMB.

#### Neuromuscular blockade

**We recommend** the use of clinical assessment and ideally complement, based on availability, with an objective monitoring by a train-of-four (TOF) peripheral nerve stimulator in adults with moderate/severe COVID-19 ARDS.

Although there is no scale to assess the level of neuromuscular blocking, the clinical practice guidelines for the sustained use of NMBs suggest guiding the titration of these drugs based on the desired clinical effect.^(33-35)^ For patients with ARDS who are compliant with mechanical ventilation and in the absence of cough before aspiration, we recommend, if available, objective monitoring of sedation depth (EEG). The TOF delivers 4 supramaximal electrical impulses to a peripheral nerve and assesses muscle fiber recruitment.^(33,35)^ The nerves commonly used are the temporal branch of the facial nerve, observing the twitch in the orbicularis oculi muscle of the eyelid or the ulnar nerve and observing the response in the abductor of the thumb. The possible score ranges from 0 to 4, with a value of 0 to 2 indicating an appropriate blockade.^(33)^ This tool should always be complemented with clinical assessment. Finally, if the patient has been administered an NMB, subjective and behavioral scale assessments of sedation and analgesia are not possible.

#### Delirium

**We recommend** using the Confusion Assessment Method for the Intensive Care Unit (CAM-ICU) to assess the presence of *delirium* in adults based on the developmental phase of COVID-19 ARDS.

**Figure 6:**
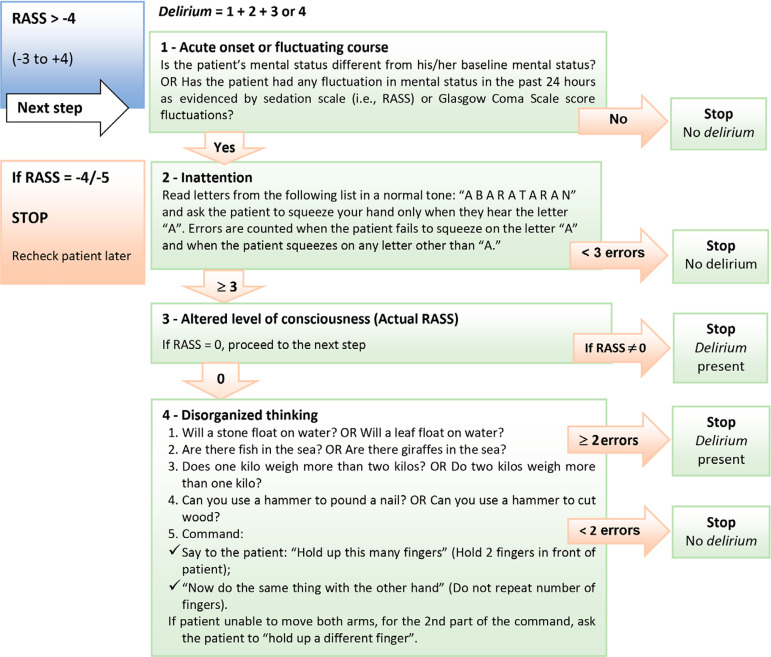
Confusion assessment method for the intensive care unit.^(38)^

The CAM-ICU assesses the 4 cardinal symptoms of *delirium*, defined as a “disturbance of consciousness characterized by acute onset and fluctuating course of inattention” accompanied by disorganized thought (Figure 6).^(36-38)^

Alternatively, the intensive care *delirium* screening checklist (ICDSC), which consists of 8 items based on the definition of *delirium* in the Diagnostic and Statistical Manual of Mental Disorders IV (DSM-IV) of the American Psychiatric Association,^(36,39)^ can be used. It takes into account more manifestations and allows better characterization of subclinical *delirium*, although some points evaluated do not have a clear operational definition. Each item receives a score of 1 if the patient clearly meets the criteria defined in the scoring instructions, while a score of 0 is given if there is no manifestation or if the item cannot be scored. *Delirium* is diagnosed based on a total score greater than 4 and a clinical assessment.^(36,39)^

### COVID-19 in a context of shortages

#### General

**We recommend** using drugs via continuous infusion, instead of intermittent administration, to reduce the number of admissions to the room and the risk of exposure to the contagion by the staff treating adults with COVID-19 ARDS.

**We recommend** developing and adopting strategies to preserve drugs of choice when possible.

**We recommend** reviewing and weighing with the Pharmacy Service the potential clinical effects of possible pharmacological interactions between drugs for analgosedation and *delirium* and the experimental drugs currently recommended as treatment support for patients with COVID-19.

The first step before starting the infusion of drugs for analgosedation is to assess the patient’s own risks and external risk factors that can alter the pharmacokinetics and pharmacodynamics of the drugs. Older adults tend to have less muscle tissue and more organ dysfunction than do younger adults, potentially leading to a decrease in metabolism and drug excretion with a risk of drug accumulation.^(40,41)^ A history of abuse of illicit drugs, opioids, antipsychotics, benzodiazepines or alcohol decreases the affinity of drugs to their receptors, with the consequent lack of efficacy at normal doses. In patients with a high body mass index, lipophilic drugs via continuous infusion, such as propofol, will lead to a risk of accumulation and prolong both desired and unwanted effects. There are also factors specific to clinical practice that can lead to changes in the choice of drug and dose adjustments; such factors include interactions between drugs administered, patient compliance with mechanical ventilation, which can lead to elevated agitation and the consequent deepening of sedation, dependence on vasopressors due to hypotension caused by sedoanalgesic drugs, etc.^(15,28,42)^

In addition to strict isolation, the risk of transmission due to exposure to secretions and aerosolization requires an aggressive approach to sedation and pain management to prevent sudden uncontrolled agitation and/or self-extubation. One review reported that the unplanned extubation rate reported in different sedation trials was up to 12%, which is unacceptably high in this context.^(28)^ One aspect to prioritize for the management of adults with COVID-19 ARDS is the use of drugs administered via continuous infusion instead of intermittently (proposed for drugs such as morphine, lorazepam, diazepam, etc.) because doing so decreases the number of entries to a room and the consequent increase in the risk of exposure to the contagion by health workers. A limitation to preparing lost-lasting infusions is evidence regarding the class of diluents, maximum dilutions, type of packaging material and environmental conditions. These conditions alter the chemical and microbiological stability of dilutions and limits the ability to make long-lasting infusions, thus increasing infusion changes and patient contact. All these aspects were taken into account in the recommendations for the dilution and administration of drugs of the *Sociedad Argentina de Terapia Intensiva* (SATI) for the Ministry of Health of the Nation: https://www.slideshare.net/SociedadArgentinadeT/covid19-dilucion-administracion-analgesicos-sedantes-bloqueantes

Due to the possible shortage of drugs of choice, which usually have a high cost and are imported, during the pandemic, it is imperative to remember and adopt drug-sparing strategies, always keeping the ABCDEF bundle of measures as a reference and avoiding the unnecessary collection of drugs in rooms so that the Pharmacy Service can manage resources based on a real-time consumption profile.^(14,43)^ Another important aspect is the possible clinical consequences of the interaction between the drugs used for analgosedation and *delirium* and the experimental drugs currently recommended as support treatment for COVID-19.^(44)^

#### Analgesia

**We recommend** maintaining an analgesia strategy first, always assessing the presence of pain and its management, before the administration or increase in sedatives in adults with COVID-19 ARDS.

**We recommend** the use of opioids for managing pain in adults with COVID-19 ARDS, regardless of disease progression. Whenever possible, we recommend evaluating the implementation of opioid-sparing strategies of choice.

First line: Fentanyl

Second line: Morphine

Alternative: Remifentanil (prioritize in the recovery phase)

One pillar of the approach is to maintain an analgesia strategy first, always assessing the presence of pain and its management, before the administration or increase in sedatives.^(35)^ Opioids continue to be the pharmacological group that has demonstrated the greatest effectiveness and safety for pain management in patients under mechanical ventilation, with intravenous drugs being the preferred options.^(15)^ An important clinical aspect to emphasize regarding fentanyl and morphine is their well-documented pharmacological interaction with benzodiazepines (midazolam and lorazepam), dexmedetomidine and propofol during their hepatic metabolism, potentially resulting in respiratory distress, hypotension and deep sedation (Table 1). There is also clinically relevant evidence for interactions between remifentanil and benzodiazepines, dexmedetomidine and propofol that can lead to episodes of hypoventilation, airway obstruction, desaturation or apnea.^(45)^ Although this evidence does not contraindicate their joint use, it is extremely important to always adjust to minimum effective doses of sedatives and opioids through an ongoing assessment of the personalized goals proposed for the analgosedation of each patient.

**Table 1:** Dose, adverse events and interactions of the proposed analgesic drugs

Drug	Dilutions Stable concentrations/compatible diluents	Initial dose and maintenance infusion	Dose adjustment	Precautions/interactions with COVID-19 management/serious adverse events
Fentanyl ampoule 250mcg/5mL	CS: in SS, maximum 20mcg/mL (in PVC or PP) In DX5%, 5mcg/mL (in PVC or PP) Pure (in PVC or PP containers) Example: 10 ampoules (2500mcg) + 100mL of SS (final volume 150mL)	Initial dose: 1mcg/kg Maintenance: CI 0.7 - 2.5mcg/kg/hour Not to exceed 10mcg/ kg/hour	Severe LF: use alternative or lower effective dose	Adverse events: hypotension - histamine release - respiratory distress Interactions: LPV/r: potential interaction, risk of accumulation. Use minimum effective dose
Remifentanil vial 5mg	Reconstitute the vial with 5mL of DW CS: in SS/DX5%, 5 - 20mcg/mL Maximum volume restriction: 400mcg/mL Caution! In RL, it is only stable for 4 hours Example: 2 vial + 100mL of SS (final volume 110mL)	Initial dose: not applicable Maintenance: CI 0.5 - 15mcg/kg/hour	RF or LF: no adjustments	Adverse events: hyperalgesia after discontinuing. Hypotension, respiratory distress Interactions: none
Morphine ampoule 10mg/1mL	CS: in SS, 0.14 - 1.5mg/mL Maximum 2.5mg/mL In DX5%, 0.1 - 1mg/mL Example: 10 ampoules (100mg) + 100mL of SS (final volume 110mL)	Initial dose: 0.05 - 0.1mg/kg Maintenance: CI 0.07 to 0.5mg/kg/hour	RF: accumulation risk Adjust: GF > 50mL/minute: 0.02 - 0.15mg/kg IV c/4 hours; GF 20 - 50mL/minute: 75% of the dose; GF: 10 - 20mL/minute: 50% of the dose LF: advised against, risk of hepatic encephalopathy	Adverse events: hypotension - histamine release Interactions: LPV/r: moderate interaction, morphine levels may decrease, implying a risk of withdrawal syndrome

CS - concentration stable for 24 hours; SS - 0.9% saline solution; DX5% - 5% dextrose; RL - Ringer's lactate; LF - liver failure; DW - distilled water; RF - renal failure; GF - glomerular filtration; CI - continuous infusion; PRIS - propofol infusion syndrome; HR - heart rate; PP - polypropylene; PE - polyethylene; PVC - polyvinyl chloride; LPV/r - lopinavir/ritonavir; HCQ - hydroxychloroquine; NA - not applicable.

Fentanyl is the most widely used drug in our context, as in the rest of the world, with a known pharmacokinetic profile, contraindications and adverse events.^(46)^ Special care should be taken when used in continuous and prolonged infusion due to its accumulation mainly in patients with severe liver failure; pain should be assessed regularly and the infusion rate periodically adjusted to achieve the lowest effective dose in these patients.^(15,45)^

Morphine is usually used to a lesser extent than fentanyl in patients under mechanical ventilation due to its lower potency, worse pharmacokinetic profile and more adverse events; however, it is an economic and well-known alternative when fentanyl is not effective or when there is a shortage.^(43)^ One of its active metabolites, morphine 6-glucuronide, accumulates in patients with kidney and liver failure; therefore, the dose must be adjusted or the infusion periodically suspended in these populations. Additionally, its administration is frequently associated with episodes of hypotension and histamine release.^(42,45,46)^ In the intermediate phase or mild ARDS, the administration of intermittent bolus morphine can be an option.

Without many other alternatives in our context, remifentanil has a rapid onset of action, does not accumulate to high levels and does not require dose adjustments in cases of kidney or liver failure; however, its high cost and fluctuating availability in our ICU, its rapid course through the body due to its pharmacokinetics and associated adverse events, such as hypotension, respiratory distress and hyperalgesia due to interrupted administration, make it a less attractive strategy for patients with COVID-19 ARDS.^(45,47)^ Therefore, remifentanil is not recommended for use as the only agent or at high doses.^(45,48)^ When possible, its use should be reserved for the recovery phase, when lighter sedation is recommended, or for brief periods.

Given the possible shortage of drugs of choice during the pandemic, a strategy that we should always evaluate and try to implement during light sedation or recovery, together with the ABCDEF bundle, is the combination of nonopioid drugs to reduce the doses of opioids of choice. The combination of pain drugs with different mechanisms of action, such as in multimodal analgesia, is important for generating synergistic effects and for reducing common opioid-related adverse events.^(42)^ However, the use of multimodal analgesia has been limited to managing postoperative pain and cancer; thus far, there is no good quality evidence for its routine use in ICUs.^(15,42,49)^ Table 2 shows the most commonly used nonopioid drugs in the ICU in case this strategy is implemented.

**Table 2:** Dose, adverse events and drug interactions for multimodal analgesia

Drug	Dilutions Stable concentrations/compatible diluents	Initial dose and maintenance infusion	Dose adjustment	Precautions/interactions with COVID-19 management/serious adverse events
Paracetamol vial 10mg/mL	Compatible with SS and DX5% CS: 1mg/mL (use immediately) Can be administered directly without prior dilution	Maintenance: 650mg every 4 hours - 1000mg every 6 hours Maximum dose ≤ 4000mg/day	LF: contraindicated in patients with severe LF RF: CrCl ≤ 30mL/minute Consider an increase in the interval between doses and a decrease in the dose	Adverse events: nausea, vomiting, headache, insomnia Interactions: none
Ketamine vial 500mg/10mL	CS: in SS, 1mg/mL (dilute 500mg in 500mL)	Initial dose: 0.1 - 0.5mg/kg Recommended: 0.15mg/kg Maintenance: CI 0.1 - 0.4mg/kg/hour Not to exceed 2mg/kg/hour	RF or LF: no adjustment required	Adverse events: psychiatric symptoms (hallucinations); respiratory distress; hypotension Interactions: LPV/r: potential interaction, risk of accumulation. Use minimum effective dose
Dexmedetomidine vial 200mcg/2mL	CS: in SS, 4mcg/mL Example: 2 vials in 100mL of SS (final volume 104mL)	Maintenance: CI 0.2 - 0.7mcg/kg/hour Recommended use for 24 hours	LF: use 0.2 - 0.7mcg/kg/hour	Adverse events: bradycardia and hypotension Moderate interaction with LPV/r (monitoring) and with HCQ Monitor the QT interval
Ketorolac ampoule 30mg/mL	CS: in SS, 0.3 - 0.6mg/mL (in PVC containers) In RL and DX5%, 0.6mg/mL (in PVC containers).	Initial dose: 30mg, then 15-30mg every 6 hours for 5 days Maximum dose: 120mg/day for 5 days Direct IV bolus, administered in no less than 15 seconds	RF: 15mg every 6 hours Maximum dose: 60mg/day	Adverse events: hypertension, edema, adverse skin reactions Interactions: None
Diclofenac ampoule 75mg/3mL	CI: mix 100mL - 500mL of SS or DX5% with an injectable solution of sodium bicarbonate (0.5mL of 8.4% solution or 1mL of 4.2% solution) Intermittent infusion: mix 1 ampoule with 50mL of SS	Maintenance: 75 mg every 12 hours Maximum recommended dose: 150mg/day	Severe LF and RF: its use is not recommended	Adverse events: renal failure, edema, cardiac arrest, skin reactions Interactions: unlikely with dexamethasone and hydrocortisone
Tramadol ampoule 50mg/mL	CS: in SS and DX5%, 0.4 - 0.5mg/mL (PVC) In RL, 0.4mg/mL (PVC)	Maintenance: 50 - 100mg every 6 hours	RF: CrCl < 30mL/minute; increase the dosing interval to every 12 hours Maximum dose: 200mg/day CrCl < 10mL/minute: 50mg every 12 hours Severe LF: 50mg every 12 hours	Adverse events: skin and gastrointestinal reactions Interactions: potential with LPV/r and with HCQ Monitor the QT interval
Carbamazepine tablet 200mg	CS: in SS and DX5%, 0.4 - 0.5mg/mL (PVC) In RL, 0.4mg/mL (PVC) NA	Initial dose: 50 - 100mg Maintenance: 100 - 200mg every 4 - 6 hours Maximum dose: 1200mg/day	Severe LF and RF: its use is not recommended	Adverse events: skin and gastrointestinal reactions, hypotension, atrioventricular block Interactions: do not administer with LPV/r and HCQ Probable interaction with dexamethasone and hydrocortisone, ivermectin and less likely with remdesivir
Gabapentin tablet 100, 300, 600mg	NA	Initial dose: 100 mg every 8 hours Maintenance: 900 - 3600mg 3 times per day	RF: CrCl 30 - 59mL/minute: 400 - 1400mg/day 2 times per day CrCl 15 - 29mL/minute 200 - 700mg/day once per day CrCl 15mL/minute: 100 - 300mg/day CrCl <15mL/minute adjust the dose in proportion to the dose for a CrCl of 15mL/minute	Adverse events: skin and gastrointestinal reactions, dizziness, drowsiness Interactions: none
Pregabalin tablet 25,50,75,150,300 mg	NA	Initial dose: 75 - 150mg Maintenance: 150 to 600mg/ day 2 times a day Usual dose: 300 - 600mg/day	RF: CrCl 30 - 60mL/minute: 75 to 300mg/day in 2 or 3 divided doses CrCl 15 - 30mL/minute 25 -150mg/day once or twice per day CrCl less than 15mL/minute 25 - 50mg once per day	Adverse events: can cause excessive sedation and hypotension. Interactions: None

CS - concentration stable for 24 hours; SS - 0.9% saline solution; DX5% - 5% dextrose; RL - Ringer's lactate; LF - liver failure; DW - distilled water; RF - renal failure; GF - glomerular filtration; CI - continuous infusion; PRIS - propofol infusion syndrome; HR - heart rate; PP - polypropylene; PE - polyethylene; PVC - polyvinyl chloride; LPV/r - lopinavir/ritonavir; HCQ - hydroxychloroquine; NA - not applicable; CrCl - creatinine clearance; QT interval - time from start of Q wave to end of T wave (electrocardiogram).

The combination of intravenous and oral opioids would likely show lower intravenous medication requirements and shorter weaning times for critically ill patients, although better evidence is needed to be able to make a favorable recommendation.^(50)^ The main barrier to using oral opioids in ICUs is their altered bioavailability due to decreased absorption by this route in critically ill patients.

#### Sedation

**We recommend** using dynamic and sequential sedation schedules according to the needs of adults with COVID-19 ARDS to avoid oversedation.

**We recommend** daily sedation “breaks” or interruptions in adults with COVID-19 ARDS only if clinical conditions specific to the patient allow and proper protection by the health team can be ensured.

**We recommend** using a pharmacological treatment schedule for sedation based on goals and disease progression in adults with COVID-19 ARDS:

Early phase or moderate/severe ARDS (deep sedation)

First line: Midazolam

Second line: Propofol

Alternative: Benzodiazepines (lorazepam and diazepam) and ketamine.

Intermediate phase or mild ARDS (light sedation) and advanced or weaning phase (light sedation or absence of sedation)

First line: Propofol

Second line: Dexmedetomidine

Alternative: Benzodiazepines (midazolam, lorazepam and diazepam) and clonidine

Patients with severe cases of COVID-19 who enter the ICU mostly present with severe hypoxemia and/or ARDS requiring mechanical ventilation, deep sedation and sometimes NMBs.^(11,51-53)^ The challenge is maintaining deep sedation strictly when necessary and, at the same time, identifying the earliest moment when light sedation can begin. It is important to recognize the benefits of avoiding deep and prolonged sedation, along with the benefits of light sedation with active participation of the family, despite not always being able to implement participation during the pandemic due to the risk of exposure and infection.^(36,54,55)^ For example, and despite its proven benefit, the application of daily sedation breaks is difficult and potentially risky in these patients. Therefore, special care must be taken, and protecting the health team should always be prioritized, even when it is detrimental to this strategy.^(56)^ Before performing a sedation break test and thus assessing light sedation, all the following criteria should be met: PaO_2_/FiO_2_ > 175mmHg, final positive end-expiratory pressure (PEEP) < 10cm H_2_O, FiO_2_ < 50%, supine for at least 4 hours, seizure-free, free of NMBs for at least 2 hours, and without extracorporeal membrane oxygenation (ECMO).^(22)^ Given that there may be a shortage of some drugs for sedation and that the average number of days that patients with severe cases of COVID-19 are mechanically ventilated is from 7 to 12 days, we recommend using dynamic and sequential schedules adjusted to each patient’s need to avoid oversedation.^(43,51)^

#### Early phase

The current guidelines for ASD under normal conditions prioritize the use of sedative drugs with short half-lives and bicompartmental pharmacokinetics and nonbenzodiazepines.^(15)^ However, in this scenario and for the above, we can prioritize drugs with longer half-lives (midazolam, lorazepam, etc.), always adjusting to minimum effective doses, which incur lower costs and are widely available in ICUs during the early period of mechanical ventilation (Table 3). The continuous infusion of ketamine in combination, within the strategies of deep sedation, can help in refractory patients to the usual treatment approach and reduce the requirements of drugs for analgosedation.^(57,58)^ A recent systematic review with meta-analysis reported that the use of ketamine as an adjuvant in an analgosedation schedule for ventilated patients would reduce requirement for propofol; however, there is uncertainty regarding clinical results, tolerance and safety profile.^(59)^

**Table 3:** Dose, adverse events and interactions of the proposed sedation drugs

Drug	Dilutions Stable concentrations/compatible diluents	Initial dose and maintenance infusion	Dose adjustment	Precautions/interactions with COVID-19 management/serious adverse events
Midazolam ampoule 5mg/mL	CS: 0.035 -1mg/mL in PCV containers and up to 2mg/mL in PP containers for SS and DX5% Not compatible with RL; can be administered pure Example: 8 ampoules (120mg) + 100 mL of SS (final volume 124mL)	Initial dose: 0.01 - 0.05mg/kg Maintenance: CI 0.02 - 0.1mg/kg/hour Not to exceed 0.2mg/kg/hour	RF: start with the lowest effective dose CrCl < 10mL/minute, reduce the dose by 50% LF: Child-pug B-C is not recommended for continuous infusion	Adverse events: respiratory distress-hypotension. Interactions: LPV/r: Potential interaction Close monitoring; use minimum effective dose
Lorazepam ampoule 4mg/mL	Stability of solution difficult Protect from light CS: in DX5%, 1 - 2mg/mL (in polyolefins or PVC) In SS, 1mg/mL in PP; 0.04mg/mL in PVC; 0.1 and 0.038mg/mL in PE In RL, 0.1mg/mL in PE Example: 10 ampoules (40mg) + 30mL DX5% (final volume 40mL)	Initial dose: 0.02 - 0.04mg/kg' (≤ 2mg) Maintenance: CI 0.01 - 0.1mg/kg/hour Infusion rate ≤ 10mg/hour	Severe RF or LF: use alternative Risk of accumulation of the excipient	Precautions: risk of accumulation of toxic excipient (propylene glycol) in renal failure, limit infusion Adverse events: respiratory distresshypotension Interactions: none
Diazepam ampoule 10mg/2mL	Stability of solution difficult Protect from light SS, 0.01mg/mL, 0.05mg/mL, 0.08mg/mL and 0.2mg/mL; DX5%, 0.04mg/mL and 0.2mg/mL Dilutions compatible in PE and glass containers Compatible with RL in glass containers CS 0.05mg/mL	Initial dose: 5 - 10mg Maintenance: 0.03 - 0.1mg/kg every 0.5 - 6 hours CI: 0.05 - 0.2mg/kg/hour	RF or LF: does not require a specific adjustment, strict monitoring due to the risk of accumulation of the excipient	Precautions: accumulation of the propylene glycol excipient can generate toxicity Strict monitoring, risk of oversedation. Adverse events: respiratory distress Interactions: Potential with LPV/r
Propofol ampoule 200mg/20mL (1%) Propofol vial 1000mg/50mL (2%)	Place the ampoules in an empty PP or PVC bag 6-hour stability Example: 4 ampoules (800mg) in empty container (final volume 80mL)	Initial dose: 5µg/kg/minute Only if hypotension is not likely Maintenance: CI 0.3 - 3mg/kg/hour Not to exceed 4.5mg/kg/hour	RF or LF: use lower doses 0.3 - 2.4mg/kg/hour	Precautions: risk of accumulation in prolonged infusions; use minimum effective dose. Adverse events: respiratory distress, hypotension, hypertriglyceridemia; risk of PRIS increases with doses greater than 3 mg/kg/h; close monitoring. Interactions: potential with LPV/r and with HCQ Monitor the QT interval
	Use directly from the vial without transferring/diluting. Once opened, 12-hour stability			
Dexmedetomidine vial 200mcg/2 mL	CS: in SS, 4mcg/mL Example: 2 vials + 100mL of SS (final volume 104mL)	Initial dose: (not recommended) 0.5mcg/kg in 15 minutes - strict HR monitoring Maintenance: CI 0.2 -1.4mcg/kg/hour	LF: monitor, use lower doses 0.2 - 0.7mg/kg/hour	Adverse events: bradycardia and hypotension Interactions: potential with LPV/r and with HCQ monitor the QT interval
Clonidine ampoule 150mcg/mL	Protect from light CS: 9mcg/mL in SS Example: 6 ampoules + 100mL of SS (final volume 106mL)	Bolus: 0.5mcg/kg (only in hemodynamically stable patients) Maintenance: CI 0.5 -2mcg/kg/hour Up to 3mcg/kg/hour	RF or LF: no adjustment	Adverse events: hypotension, bradycardia, atrioventricular block Interactions: none

CS - concentration stable for 24 hours; SS - 0.9% saline solution; DX5% - 5% dextrose; RL - Ringer's lactate; LF - liver failure; DW - distilled water; RF - renal failure; GF - glomerular filtration; CI - continuous infusion; PRIS - propofol infusion syndrome; HR - heart rate; PP - polypropylene; PE - polyethylene; PVC - polyvinyl chloride; LPV/r - lopinavir/ritonavir; HCQ - hydroxychloroquine; NA - not applicable; CrCl - creatine clearance.

Diazepam has a long half-life and is almost exclusively metabolized in the liver, generating active metabolites that have a very high risk of accumulating in patients with impaired kidney function.^(45)^ Like lorazepam, diazepam should be administered as a slow infusion, is prone to causing *delirium* and has an excipient (propylene glycol) that accumulates in patients with renal failure and can be very toxic, leading to metabolic acidosis and kidney damage. There is no good quality evidence on its continuous infusion for critical patients; therefore, it should be used exclusively during shortages of usual drugs, and very prolonged infusions should be avoided.^(45,58)^ Finally, the risk of oversedation is substantial with these drugs; therefore, their dosage and monitoring should be strictly assessed.

#### Intermediate and advanced phases

In the recovery phase, with more light sedation and without the need for NMB agents and prone positioning sessions, we can prioritize drugs with more favorable pharmacokinetics, shorter half-lives, and less accumulation (propofol and dexmedetomidine).^(42,60)^ If the hemodynamic situation allows, the use of propofol alone or in combination with benzodiazepines (usually midazolam) should be considered because this approach has been shown to lead to shorter ICU stays and a lower incidence of *delirium* than benzodiazepines alone. However, for continuous propofol infusion, constant monitoring is necessary due to the risk of respiratory distress, hypotension, hypertriglyceridemia and, after prolonged periods of infusion and the maximum dose, the appearance of propofol infusion syndrome (PRIS).^(45,61)^

The combination of dexmedetomidine with other analgesic drugs has been shown to reduce the dose of midazolam, propofol and opioids.^(62)^ Compared with propofol, dexmedetomidine is associated with a shorter ICU stay and a lower incidence of *delirium*, while in patients with prolonged mechanical ventilation, it can reduce the number of days on mechanical ventilation and keep patients in a communicative state.^(63-65)^ An alternative to dexmedetomidine is clonidine; however, the evidence supporting its use in the setting of critical patients is scarce and of low quality; therefore, it should only be utilized in cases of shortages in usual therapies.^(65)^ Its alpha-2 effect (like dexmedetomidine), its low cost and its adequate safety profile in hemodynamically stable patients make it an attractive alternative when dexmedetomidine is not an option. In turn, in patients receiving dexmedetomidine, during the weaning phase, a gradual transition is recommended to avoid withdrawal and anxiety events prior its discontinuation.^(66-68)^

Although the use of ketamine is not very widespread in our ICU, its use can be useful as an alternative in light sedation and, due to its analgesic properties, as an opioid- or benzodiazepine-sparing strategy when combined with those drug options.^(69,70)^ The administration of ketamine is associated with frequent and serious adverse neurological events, such as respiratory distress and hypotension, which limit the recovery of patients with COVID-19 ARDS. It is not recommended for patients with suspected unstable angina, uncontrolled high blood pressure or intracranial hypertension. Importantly, assessing anesthetic depth with BIS^©^ loses validity and correlation for patients receiving ketamine.

When the respiratory picture of a patient is maintained for 48 hours (PaO_2_/FiO_2_ > 200, FiO_2_ < 60% and PEEP < 15 cm H_2_O), without NMBs and without requiring prone decubitus positioning, it is possible to proceed to shorter half-life drugs, with opioid-sparing strategies. In this third stage, the goal is to achieve a RASS of 1 to -1, with special attention to the adequate pharmacological and nonpharmacological management of pain and anxiety and the daily monitoring of the presence of *delirium*. If dexmedetomidine was used in previously sedated patients, induction should not be performed, given that it will take 6 hours to reach the desired effect. If induction is used, 0.5mcg/kg in 15 minutes with heart rate monitoring, because transient hypertension may occur, is recommended.

#### Neuromuscular blocking agents

**We recommend** using neuromuscular blocking agents, starting with intermittent infusion, only in precise clinical conditions of the patient, and according to goals and disease progression of adults with COVID-19 ARDS:

Early phase or moderate/severe ARDS (deep sedation)

First line: Atracurium

Second line: Vecuronium or rocuronium

Alternative: Pancuronium

Patients with COVID-19 ARDS require prolonged mechanical ventilation and deep sedation, usually associated with the continuous use of NMBs, resulting in a high risk of sequelae during and after their ICU stay.^(71)^ The use of NMBs is recommended for patients with severe ARDS, with PaO_2_/FiO_2_ < 150 despite an optimal ventilatory strategy and in whom adequate mechanical ventilation compliance is not achieved despite having reached RASS -4/-5; when increasing the levels of sedation is not recommended.^(35,72,73)^

Regarding the clinical benefit of this strategy in the early stages of ARDS, there is contradictory evidence regarding improvements in clinical outcomes in this population, although it can help to limit patient self-inflicted lung injury (P-SILI) and lung injury associated with patient-ventilator asynchronies due to double triggering and reverse triggering, and can avoid aerosolization.^(22,74-76)^ In patients who decide to use NMBs (for example, severe ARDS with prone ventilation), we recommend nondepolarizing blockers with intermittent bolus infusions to facilitate lung protective ventilation and the prone position, only moving to continuous infusion if there is persistent asynchrony or severe hemodynamic compromise, reassessing every 24 hours.^(22,77)^

The clinical practice guidelines and consensus of specialists worldwide recommend cisatracurium as the first choice for patients with ARDS, also being the most studied in this population.^(15,43,76)^ Because it is not available in Argentina, our first choice recommendation is atracurium. Atracurium, like cisatracurium, has a benzylisoquinoline structure and is an intermediate-acting NMB that is metabolized by plasma esterases and Hofmann elimination, which favors its use in patients with kidney or liver failure (Table 4).^(76,78)^ However, due to its histaminergic effect, it can produce an increase in respiratory secretions and, with prolonged use, the accumulation of laudanosine, a potentially neurotoxic metabolite.^(45,73)^

**Table 4:** Dose, adverse events and interactions of the proposed drugs for neuromuscular block

Drug	Dilutions Stable concentrations/compatible diluents	Initial dose and maintenance infusion	Dose adjustment	Precautions/interactions with COVID-19 management/serious adverse events
Atracurium ampoule 50mg/5mL	CS: in SS/DX5%. 0.2 - 1mg/mL Max 5mg/mL; incompatible with RL Example: 10 ampoules (500mg) + 100mL of SS (final volume 150mL)	Initial dose: 0.4 - 0.5mg/kg Maintenance: 5 - 20mcg/kg/minute	RF or LF: no adjustment required	Adverse events: risk of histamine release (minimum) Tachyphylaxis (if continuous infusion is prolonged over time) Interactions: none
Vecuronium vial 10mg	Reconstitute each vial with 10mL of distilled water Compatible with SS, DX5%, RL Example: 5 ampoules (50mg) + 100mL of SS (final volume 150mL)	Initial dose: 0.08 - 0.1mg/kg Maintenance: 0.8 - 1.7mcg/kg/minute	RF or acute emergency RF: use minimum effective dose due to accumulation risk	Adverse events: vagal blockage with high doses Interactions: none
Rocuronium ampoule 50mg/5mL	Compatible with SS, DX5% and RL CS: SS, RL and DX5%, 0.5 and 2mg/mL Example: 4 ampoules (200mg) + 100mL of SS (final volume 120mL)	Initial dose: 0.06 - 1mg/kg Maintenance: 8 - 12mcg/kg/minute	RF or LF: no adjustment required, assess dose-response	Adverse events: bradycardia, vagal blockage with high doses Interactions: potential with LPV/r or with HCQ, monitor the QT interval
Pancuronium ampoule 4mg/2mL	Compatible with SS, DX5%, RL Example: 10 ampoules (40mg) + 100mL of SS (final volume 120mL)	Initial dose: 0.04 - 0.1mg/kg Maintenance: 1 - 2mcg/kg/minute	RF: use minimum effective dose, due to risk of accumulation Avoid its use in patients with severe RF, CrCl <10mg/mL	Adverse events: respiratory distress, hypertension, vagal blockage with high doses Interactions: none

CS - concentration stable for 24 hours; SS - 0.9% saline solution; DX5% - 5% dextrose; RL - Ringer's lactate; LF - liver failure; DW - distilled water; RF - renal failure; GF - glomerular filtration; CI - continuous infusion; PRIS - propofol infusion syndrome; HR - heart rate; PP - polypropylene; PE - polyethylene; PVC - polyvinyl chloride; LPV/r - lopinavir/ritonavir; HCQ - hydroxychloroquine; NA - not applicable

For the second line, vecuronium or rocuronium, with intermediate-acting effects, can be considered and can also be used via continuous infusion. These drugs are metabolized in the liver and can lead to kidney and liver failure, but their advantage over pancuronium is that there is an antidote (sugammadex) that quickly reverses the neuromuscular blockade. Pancuronium, being long-acting, is an alternative with more unfavorable pharmacokinetics that can be considered for continuous or intermittent administration.^(35)^

#### Algorithm for analgosedation in adults with COVID-19 ARDS in the context of shortages

**Figure 7:**
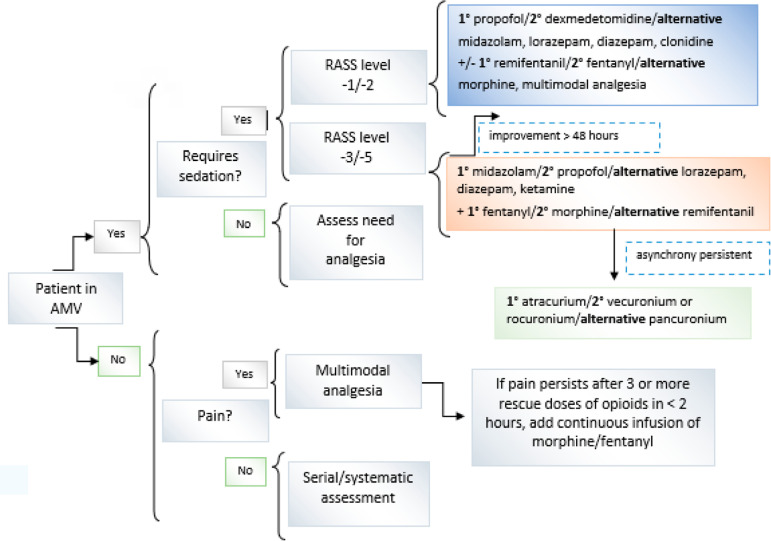
Proposed algorithm for analgosedation of adults with COVID-19 acute respiratory distress syndrome in the context of drug shortages.

Figure 7 shows a proposed algorithm, following the usual recommendations, with the goal of preserving the drugs of choice, reducing agitation and/or *delirium* and facilitating the removal of mechanical ventilation with the maximum physical and cognitive well-being possible. It is likely that in the face of the COVID-19 pandemic, it will be necessary to use modified schedules due to shortages in drugs of choice, medical devices, or health personnel, which can lead to prolonged protective mechanical ventilation times due to severe ARDS and slower recovery processes.^(22)^ This document will consider the possible deviations of the proposed algorithm and will present alternatives to resolve them.

AMV - assisted mechanical ventilation; RASS - Richmond Agitation-Sedation Scale.

### Comprehensive therapeutic approach for patients with delirium in the intensive care unit

**We recommend** not using routine pharmacological treatment for the prevention or management of *delirium* in adults with COVID-19 ARDS.

**We recommend**, if possible, adopting nonpharmacological measures, such as early comfort using analgesia, minimal sedatives and maximal human care (eCASH), to prevent and decrease *delirium* in adults with COVID-19 ARDS.

*Delirium* results from acute organ failure and is characterized by the acute onset of altered consciousness and attention with a fluctuating course. Importantly, its appearance in patients admitted to the ICU is associated with a higher mortality rate.^(79)^ It should be borne in mind that there is no pharmacological treatment that has proven effective for managing *delirium*; therefore, prevention is essential.^(15)^*Delirium* is especially high in mechanically ventilated patients with COVID-19, reaching up to 75%, and confusion was the fifth most frequent characteristic of these patients in the United Kingdom.^(80-82)^ Taking into account predisposing factors (frailty, cognitive decline, etc.) and precipitating factors (which can be classified into 3 domains: disease severity, exposure to medications and environmental factors), it is likely that this increase in prevalence of *delirium* is not a product of a specific brain tropism of the virus but rather a massive burden of precipitating factors (regarding sedation, immobility, isolation, etc.).^(81,83)^ The concept of eCASH proposed by Vincent et al. is a good starting point for discussing nonpharmacological treatment measures.^(54)^ Comfort as a first need, adequate analgesia, minimal sedation and humanized care focused on the patient and family are the axes of this new paradigm for intensive medicine. Based on this concept, the ABCDEF bundle can be understood as “the way” to achieve this ideal.^(14)^

Similar to the PADIS clinical practice guidelines, we do not recommend, due to a lack of evidence and clinical benefit, routinely using haloperidol, typical or atypical antipsychotics or other drugs to prevent or manage *delirium*.^(15,84-89)^ In case it is decided to use this strategy, we suggest using it for agitated patients (RASS > 1) and at low doses and prioritizing short half-life drugs and lower accumulation risk (Table 5). The use of physical restraint should be used only under exceptional circumstances, taking into account that it does not prevent adverse effects, is traumatic for the patient and aggravates *delirium*. In the MENDS study (Maximizing Efficacy of Targeted Sedation and Reducing Neurological Dysfunction), the use of dexmedetomidine resulted in more *delirium*-free or coma-free days and more time in sedation compared to the use of lorazepam in patients under mechanical ventilation.^(90)^ These effects have also been reported in a comparison of this drug with propofol or midazolam.^(91)^ In the case of mechanically ventilated agitated patients who cannot be extubated due to agitation, the use of dexmedetomidine could be useful.^(92)^ Finally, as we have previously mentioned, the key lies in the use of organized bundles of measures (ABCDEF) and not in the use of a single drug.^(93)^

**Table 5:** Dose, adverse events and interactions of the proposed drugs for *delirium*

Drug	Dilutions Stable concentrations/ compatible diluents	Initial dose and maintenance infusion	Dose adjustment	Precautions/interactions with COVID-19 management/serious adverse events
Quetiapine tablets 25, 50, 100, 200mg	NA	Maintenance: 50mg every 12 hours (maximum dose of 200mg every 12 hours)	LF: start at 25mg/day	Adverse events: prolongation of the QT interval, hypertension, tachycardia Interactions: LPV/r: Potential interaction. Could increase the concentration of quetiapine
Olanzapine tablets 2.5, 5, 10mg	NA	Maintenance: 5mg/day	RF: no adjustment LF: no adjustment	Adverse events: orthostatic hypotension, peripheral edema, hypercholesterolemia Interactions: unlikely with LPV/r
Risperidone tablets 0.5, 1, 2, 3, 4mg	NA	Maintenance: 0.5mg every 12 hours (maximum dose 4mg/day)	Severe RF: use minimal doses Severe LF: use minimal doses	Adverse events: prolonged QT interval, blood dyscrasias Interactions: LPV/r: potential prolongation of the QT interval; HCQ: potential increase in risperidone concentration
Haloperidol ampoules 5mg/mL	CS: 0.1mg/mL DX5% in glass container Not tested in other dilutions or containers	Maintenance: 2.5mg every 8 hours (maximum dose 20mg/day)	In older adults, use minimal doses	Adverse events: prolongation of the QT interval, hypotension, Torsades de pointes Interactions: do not administer with LPV/r or HCQ
Haloperidol tablets 1, 5, and 10mg	NA	Maintenance: 2.5 to 5mg every 8 hours	In older adults, use minimal doses	Adverse events: prolongation of the QT interval, hypotension, Torsades de pointes Interactions: do not administer with LPV/r or HCQ

CS - concentration stable for 24 hours; SS - 0.9% saline solution; DX5% - 5% dextrose; RL - Ringer's lactate; LF - liver failure; DW - distilled water; RF - renal failure; GF - glomerular filtration; CI - continuous infusion; PRIS - propofol infusion syndrome; HR - heart rate; PP - polypropylene; PE - polyethylene; PVC - polyvinyl chloride; LPV/r - lopinavir/ritonavir; HCQ - hydroxychloroquine; NA - not applicable.

### Early mobility and family engagement

**We recommend**, if possible, maintaining at least one passive motion from the initiation of mechanical ventilation and adopting protocols that allow including the entire work team and family of adults with COVID-19 ARDS.

The exposure of health personnel and the risk of infection make the proper use of PPE (N95-type mask, isolation gown, gloves, eye protection and face mask), adequate hand hygiene and minimizing all potential infection risks, such as self-extubation and agitation, among others, a priority. An example is the first wave of infections in the Italian Lombardy region, where approximately 9% of SARS-CoV-2 infections were in health care workers.^(94)^ Thus, conflict arises between what is proposed by the ABCDEF bundle and the concept of eCASH (alert and calm patient accompanied by their family) and the conjuncture posed by the pandemic with patients requiring protective mechanical ventilation, deep sedation in many cases, and strict isolation for both the family and the treatment team.^(20,54)^ As a result of the increased risk of infection in the ICU and to preserve the health of work teams and patients, many centers have preemptively reduced the entry of health personnel, such as kinesiologists, occupational therapists, psychologists, and social workers, and families. As epidemiological and ICU conditions improve, we believe it is essential to prioritize the reincorporation of the entire health care team through simple and clear protocols.

With respect to early mobility, it is important to maintain at least passive motion from the initial moment of mechanical ventilation, assessing the feasibility of advancing in that process as the clinical situation of the patient allows. The early mobility of mechanically ventilated patients by a multidisciplinary team has proven to be a feasible, safe procedure and has been shown to have a clinically relevant impact on pre-pandemic conditions.^(95-97)^ In a recent study, flexible family engagement as proposed in the ABCDEF bundle, when compared to a more restrictive regimen, did not affect clinical outcomes in patients and staff but did reduce anxiety and depression in their relatives.^(98)^ Despite this result, the pandemic and isolation, in daily practice and according to the experience of patients and families, we believe it is necessary to bring patients closer to their families. Before the implementation of these measures, for Argentina, we recommend reading the law on telecare and the provision of legal advice, to always protect the rights of patients and comply with the professional responsibility of the work team.^(99)^

## DISCUSSION

The management of ASD in critical patients has changed profoundly in the last 20 years, going from mostly deep-sedated patients to patients with lighter sedation targets, better pain management, early mobility and family engagement as part of treatment. However, the pandemic forced situations involving noncooperative patients who were polymedicated and isolated from their families and health personnel. This consensus advances the guidelines for analgosedation in critical patients with COVID-19 ARDS performed by experts with the best available evidence in high-income countries but under the experience and perspective of the situation of ICUs in our country and Latin America.^(22,43,58,71,77,83,100)^

A limitation that results from adapting international guidelines to our context is the prioritization in certain phases of drugs already relegated in treatment protocols. This measure was mainly conceived as a strategy for sparing drugs of choice, which in our country are typically costly and usually imported. However, the drugs prioritized in this consensus are present in relevant international clinical practice guidelines and, when appropriately used, as detailed in this document, can be great allies to safeguard drugs of choice and still generate clinical benefits in patients.^(15,101)^

Faced with the new reality in ICUs created by the ongoing COVID-19 pandemic, we should not abandon the usual “good practices” but adapt them. This crisis should be used as an opportunity to implement a systematic approach based on the best available evidence, prioritizing targeted strategies with adequate pain control and a progressive reduction in sedation and its adverse effects in the short and medium terms. Likewise, it will allow us to adapt the system in case of health resource scarcity resulting from the pandemic. The performance of the multidisciplinary team inside and outside the ICU and their ability to identify, assess and adapt protocols based on the best available evidence, even before authorities at the regional or national level can incorporate changes in the general protocols, are examples of the versatility and commitment of that change.

Our consensus has the ultimate goal of solving these problems that the pandemic commonly poses in our region; therefore, we consider it appropriate to divide disease progression into different stages to plan ASD management for patients on mechanical ventilation. Regardless of the drugs used, we believe that it is essential that each ICU design its own management schedules for sedation, analgesia, *delirium*, mobility and family engagement to achieve a consistent approach in the management of its patients and thus improve clinical outcomes.^(102)^
